# Dysphonic Voice Pattern Analysis of Patients in Parkinson's Disease Using Minimum Interclass Probability Risk Feature Selection and Bagging Ensemble Learning Methods

**DOI:** 10.1155/2017/4201984

**Published:** 2017-05-03

**Authors:** Yunfeng Wu, Pinnan Chen, Yuchen Yao, Xiaoquan Ye, Yugui Xiao, Lifang Liao, Meihong Wu, Jian Chen

**Affiliations:** ^1^School of Information Science and Technology, Xiamen University, 422 Si Ming South Road, Xiamen, Fujian 361005, China; ^2^Department of Rehabilitation, Zhongshan Hospital, Xiamen University, 201 Hubin South Road, Xiamen, Fujian 361004, China

## Abstract

Analysis of quantified voice patterns is useful in the detection and assessment of dysphonia and related phonation disorders. In this paper, we first study the linear correlations between 22 voice parameters of fundamental frequency variability, amplitude variations, and nonlinear measures. The highly correlated vocal parameters are combined by using the linear discriminant analysis method. Based on the probability density functions estimated by the Parzen-window technique, we propose an interclass probability risk (ICPR) method to select the vocal parameters with small ICPR values as dominant features and compare with the modified Kullback-Leibler divergence (MKLD) feature selection approach. The experimental results show that the generalized logistic regression analysis (GLRA), support vector machine (SVM), and Bagging ensemble algorithm input with the ICPR features can provide better classification results than the same classifiers with the MKLD selected features. The SVM is much better at distinguishing normal vocal patterns with a specificity of 0.8542. Among the three classification methods, the Bagging ensemble algorithm with ICPR features can identify 90.77% vocal patterns, with the highest sensitivity of 0.9796 and largest area value of 0.9558 under the receiver operating characteristic curve. The classification results demonstrate the effectiveness of our feature selection and pattern analysis methods for dysphonic voice detection and measurement.

## 1. Introduction

Dysphonia is a phonation disorder with the difficulty in the voice production. Dysphonia can be observed with hoarse, harsh, or breathy vowel sounds, as a result of impaired ability of the vocal folds to properly vibrate during exhalation [1]. Idiopathic Parkinson's disease (IPD) is known as a chronic neurodegenerative disorder that may lead to producing dysphonic voice due to probable neurogenic interruptions in the laryngeal nerve paths [2]. It is reported by Sewall et al. [2] that about 70% to 80% of IPD patients would suffer from dysphonia or other phonatory disorders, with the symptoms of decreased variation, roughness, increased asthenia, dysarthria, or voice tremor. The neurological dysfunction and debilitated communicative deficits of IPD patients greatly cause impact on their social communications and quality of life [3].

Characterization and quantification of vocal parameters are useful for better understanding of the perceptual changes in phonation system of IPD patients in accordance with the clinical disease progression [4, 5]. Impaired vocal folds and articulatory and fluency deficits of IPD patients may result in altered vibrations of the glottis, changes of acoustic amplitude, and pitch frequency variations, when producing vowel sounds. Recent studies [4–8] have attempted to quantify dysphonic voice parameters such as acoustic amplitude and frequency variations, with the purpose of characterizing the IPD dysphonic symptoms. Holmes et al. [6] examined the perceptual voice characteristics of IPD patients with different disease stages. They compared the perceptual and acoustic characteristics between 60 IPD patients and 30 normal control subjects. Their results showed that IPD has significant manifestations of loudness variability, lower maximum phonation frequency, breathiness, harshness, and reduced loudness [6]. Goberman et al. [9] investigated the acoustic characteristics of fundamental frequency (*F*0) variability in vowels, mean of *F*0, and intensity range of IPD patients. They reported that the jitter and mean *F*0 values would increase and the intensity range would become smaller in IPD patients than in healthy subjects. Rahn et al. [10] and Vaziri et al. [11] also computed some fractal and entropy parameters of IPD patients and control subjects, in order to measure the nonlinear dynamics of sustained vowel sounds in their speech tests. Their experiments demonstrated a significant increased acoustic signal complexity in terms of fractal dimensions and period entropies can be observed in IPD patients with voice impairment and phonation disorders [10, 11].

With a number of phonatory frequency variability, variation of speech amplitude (shimmer), intensity, and nonlinear dynamics parameters computed from the electroglottographic signals in standard speech tests, it is necessary to select the most discriminant vocal parameters with feature combination methods for further pattern classifications.

Filter methods for feature selection or combination are usually less computationally intensive than the wrapper methods that commonly use a predictive model to score feature subsets [12]. Plenty of statistical feature filter methods are computed based on probability distribution estimations [5, 12]. The mutual information gain, interclass distance based on estimated probability densities, or the scores of significance tests are widely used measures to filter the optimal feature subsets with the filter methods [5]. In order to measure the information gain of each feature, the Kullback-Leibler divergence (KLD) [13] can be utilized to calculate the interclass distance of feature probability densities between the healthy controls and patients with Parkinson's disease. The Kullback-Leibler divergence is the information gained when one revises one's beliefs from the prior probability distribution to the posterior probability distribution; that is, the KLD measures the amount of information lost when the prior probability distribution is used to approximate the posterior probability distribution [13]. However, the KLD proposed by Kullback and Leibler [14] is a type of nonsymmetric metric that estimates the relative entropy of the posterior probability distribution over the prior probability distribution [15]; Wu and Shi suggested a revision of the KLD (named the modified Kullback-Leibler divergence, MKLD) with the symmetry divergence adjustment, to better characterize the gait feature probability densities between healthy adults and amyotrophic lateral sclerosis [16]. It is worth noting that either KLD or MKLD commonly leads to the systematic bias, because the prior probability density in the relative entropy denominator sometimes has to be revised to avoid a value of zero.

The aim of the present work is to propose a novel probabilistic-based class-confusion information measure method, by means of estimating the overlapping area of the feature probability distributions between two classes. It is hypothesized that the combination of the selected vocal features based on the interclass probability risk rule could provide more discriminative information for pattern analysis. In addition, we plan to study the correlations between multiple vocal parameters and also develop the methods of feature computing and analysis of vocal patterns. It is hypothesized that the advanced machine learning algorithms with the selected multivariate features may effectively distinguish the vocal patterns between patients with Parkinson's disease and healthy control subjects.

The rest of the paper is organized as follows.  Section 2 describes the voice data set and related acoustic features for further pattern analysis.  Section 3 describes the feature selection and pattern classification methods used in our computer experiments.  Section 4 presents the results of feature correlations, feature selection, and pattern classifications in detail, along with the result analysis and limitation discussions.  Section 5 concludes the present study and provides the perspective on future possible related works.

## 2. Materials

### 2.1. Voice Data Set

The phonation data tested in the present work were provided by Little et al. [7] for public research usage and can be online accessed via the University of California at Irvine machine learning repository [17]. The data set consists of 195 sustained vowel voice records phonated by a total of 31 subjects (48 vowel phonations recorded from the subjects in the normal group and 147 vowel phonations recorded from the patients with Parkinson's disease in the pathological group). The normal group involves 8 healthy control (CO) subjects (3 males and 5 females, age mean ± standard deviation, SD: 60.2 ± 8.6 years). The pathological group contains 23 IPD patients (16 males and 7 females, age mean ± SD: 67.8 ± 9.7 years). The disease stages of IPD symptoms were rated using the modified Hoehn and Yahr (MHAY) scale [18], a most commonly used IPD progress assessment method in neurological diagnosis, for 23 IPD patients. Details of the MHAY stages of the IPD patients are listed in Table 1. It is noted that 17 (73.9%) IPD patients were with mild, moderate, and even severe functional impairments (MHAY > 2).

The speech recording experiments were carried out by Little et al. [7], with all 31 subjects providing their written informed consent reviewed and approved by University of Oxford, United Kingdom, and United States National Center for Voice and Speech, Denver, Colorado. Each subject was requested to pronounce vocal vowels with a head-mounted microphone positioned at 8 cm in front of the lips. The head-mounted microphone was calibrated by using a Class 1 sound level meter (Brüel & Kjær Type 2238 Mediator) [7]. The acquired acoustic signals were sampled at 44.1 kHz with 16-bit resolution per sample. The amplitude of each signal was digitally normalized in order to suppress the effects of individual difference [7]. The Kay Pentax multidimensional voice program (MDVP) was used by Little et al. [7] to measure 16 voice perturbation parameters, including the period (jitter) and amplitude (shimmer) perturbations, and harmonics-to-noise (and noise-to-harmonics) ratios. Six additional nonlinear parameters were also computed by Little et al. [7] to characterize the signal complexity degree and fractal dimensions of the dysphonic voice records. For more details on speech recording protocol and acoustic signal acquirement experiments, please refer to the related work of Little et al. [7].

### 2.2. Feature Descriptions

There are a total of 22 vocal features available in the phonation data set provided by Little et al. [7]. Details of the feature description are listed in Table 2. For the convenience of voice perturbation feature presentation, we named the average, maximum, and minimum vocal fundamental frequency (in Hz) computed by the Kay Pentax multidimensional voice program (MDVP) with the abbreviations of MDVP:F0, MDVP:Fhi, and MDVP:Flo, respectively. The percentage and absolute jitter values are expressed as MDVP:Jitter(%) and MDVP:Jitter(Abs). The five-point period perturbation quotient and relative amplitude perturbation parameters calculated by the MDVP are written as MDVP:PPQ and MDVP:RAP. The Jitter:DDP denotes the average absolute difference of differences between jitter cycles. The original and logarithmic units of the MDVP local shimmer parameter are named MDVP:Shimmer and MDVP:Shimmer(dB). The abbreviations of Shimmer:APQ3 and Shimmer:APQ5 are short for the three-point and five-point shimmer perturbation quotient values, respectively. MDVP:APQ11 represents the 11-point amplitude perturbation quotient value. Shimmer:DDA is the average absolute differences between the amplitudes of consecutive periods. The noise-to-harmonics ratio and harmonics-to-noise ratio of the acoustic signals are abbreviated as NHR and HNR, respectively. Several nonlinear features include the correlation dimension (D2), recurrence period density entropy (RPDE), detrended fluctuation analysis (DFA), and pitch period entropy (PPE). Two nonlinear measures of fundamental frequency variation are presented as Spread1 and Spread2, respectively.

### 2.3. Feature Correlation Analysis

It is noted that several vocal features characterize the similar perturbation and nonlinear properties; for example, Shimmer:APQ3, Shimmer:APQ5, and MDVP:APQ11 characterize the amplitude variations. It is therefore necessary to analyze the vocal feature correlations in order to minimize the similarity redundancy [5]. In the present study, we computed the correlation coefficients between the vocal feature pairs. The strong linear correlation relationship between each feature pair was empirically defined with the Pearson correlation coefficient over 0.8.

## 3. Methods

### 3.1. Feature Selection

With a number of fundamental frequency perturbation, amplitude variation, and nonlinear signal dynamics features at hand, we considered selecting the most representative feature combination for further pattern analysis. In this work, we applied the Parzen-window method to establish the probability density function (PDF) of each feature for the IPD and CO subject groups, respectively.

The Parzen-window method is one of the nonparametric kernel-based PDF modeling techniques, which can be used to establish multimodal PDFs [19, 20]. The Parzen-window method commonly estimates an unknown PDF by averaging the accumulated nonnegative kernel functions *κ*(·), the centers of which are located at the vocal pattern data points *x*_*i*_, written as

(1) $$\begin{array}{c} \hat{f}\left(\;x\;\right)=\frac{1}{N\;h}\overset{N}{\underset{n=1}{\sum }}\kappa \left(\;\frac{x-x_{i}}{h}\;\right), \end{array}$$

where *N* is the number of data points and *h* represents the kernel bandwidth. In the present study, the Gaussian radial basis function was chosen as the kernel window function. According to Hollander et al. [12], the optimal kernel bandwidth of the Gaussian function is given by

(2) $$\begin{array}{c} h_{opt}=1.06\times SD\times N^{-1/5}, \end{array}$$

where SD denotes the standard deviation of the data points.

Based on the estimated PDFs of each vocal feature for the IPD and CO groups, we would like to analyze and select the possible feature combinations that may contain the most representative discriminant information on pattern classifications. We first calculated the modified Kullback-Leibler divergence (MKLD) to compare the feature differences between the IPD and CO subject groups. The MKLD is revision of the Kullback-Leibler divergence to make a symmetry adjustment of such a relative entropy measure between the probability distributions of ${\hat{f}}_{IPD}(x)$ and ${\hat{f}}_{CO}(x)$ for two subject groups [16], which can be written as

(3) MKLD=∫−∞∞f^IPDxln⁡f^IPDxf^COxdx+∫−∞∞f^COxln⁡f^COxf^IPDxdx.

If two probability distributions are similar or completely the same, the MKLD value is close to zero. On the other hand, the MKLD value would become large, if two classes are discriminant based on their probability distributions.

The MKLD is better than the Kullback-Leibler divergence (KLD) because the MKLD is a symmetric probability density measure metric that calculates the twofold relative entropy values between two feature probability distributions. However, it can be observed from the MKLD definition that the relative entropy should avoid a zero denominator with minor numeric revisions, such that the MKLD feature divergence computing sometimes would bring in the systematic bias. With the purpose of better representing the probability density differences of the distinct vocal features, in this work, we propose an overlapping feature distribution measure method to estimate the probabilistic confusion between two classes. The interclass probability risk (ICPR) is computed with the integration of the overlapped PDFs as

(4) $$\begin{array}{c} ICPR=\overset{\infty }{\underset{-\infty }{\int }}min\left[\;{\hat{f}}_{IPD}\left(\;x\;\right),{\hat{f}}_{CO}\left(\;x\;\right)\;\right]dx. \end{array}$$

If the entire feature probability distributions of two classes are overlapping, the value of ICPR is equal to 1. When two classes are completely separable without any PDF overlap, the ICPR value becomes zero. In general, if the ICPR value is smaller, the classes are easier to be separated with the given feature distributions. The feature selection based on the ICPR measure has a major advantage that it can be adaptive to unimodal or multimodal probability densities.

In order to determine the best feature combination for further pattern classifications, the ICPR and MKLD measures were used as the feature selection metrics in our experiment, respectively. If the probability densities of two classes are overlapped at a random guess level, that is, the overlapped area of two probability densities is equal to the resting nonoverlapped probability density areas of both two class, the ICPR value is about to be 0.67. The optimal features selected by the ICPR method are MDVP:F0, Spread1, MDVP-LDA, Shimmer-LDA, and Nonlinear-LDA, with the ICPR value lower than 0.6, the features of which could help the classifiers make a decision better than a random guess. Since the MKLD is a symmetry metric with the sum of a pair of KLDs, the probability densities of two classes that are overlapped at a random guess level would produce a MKLD value of 1. In our experiments, the best features selected by the MKLD method are MDVP:F0, MDVP:Flo, MDVP-LDA, Shimmer-LDA, and Nonlinear-LDA, with the MKLD value larger than 1.

### 3.2. Pattern Classifications

Based on the selected features with the ICPR and MKLD methods, we used three different nonlinear classification methods, that is, generalized logistic regression analysis (GLRA), support vector machine (SVM), and Bagging ensemble algorithm, to distinguish the voice patterns based on the selected feature set. The voice pattern classes of the healthy control subjects and IPD patients were assigned with the negative label (−1) and positive label (+1).

#### 3.2.1. Generalized Logistic Regression Analysis

As an extension version of binary logistic regression analysis, the generalized logistic regression analysis establishes a multinomial logistic model in order to describe the systematic relationship between the multivariate feature inputs and the explanatory outcome. The generalized logistic regression analysis model also contains a random component with the Bernoulli distribution to characterize the stochastic effects [21]. The logit link function of generalized logistic regression analysis calculates the natural logarithm of an odds ratio of the binomial probabilities, which can be written as

(5) $$\begin{array}{c} ln\left(\;\frac{P_{IPD}}{P_{CO}}\;\right)=x^{T}\beta =\beta_{0}+x_{1}\beta_{1}+\cdots +x_{f}\beta_{f}, \end{array}$$

where *P*_IPD_ and *P*_CO_ = 1 − *P*_IPD_ denote the probabilities of binary classes (i.e., IPD and CO subject groups), the vector **β** = [*β*_0_, *β*_1_,…, *β*_*f*_]^*T*^ represents the generalized logistic regression coefficients, and **x** = [1, *x*_1_,…, *x*_*f*_]^*T*^ is the model input vector including unity and five selected vocal features, the latter of which include MDVP:F0, Spread1, MDVP-LDA, Shimmer-LDA, and Nonlinear-LDA. The regression coefficients were calculated with the maximum likelihood estimation by following the iterative weighted least-squares procedure [22]. The optimal generalized logistic regression coefficients were estimated to be **β**_opt_ = [1.362, −0.003,7.856, −0.021,0.005,25.71]^*T*^, which could make the generalized logistic regression analysis model to achieve the largest area under the receiver operating characteristic (ROC) curve.

#### 3.2.2. Support Vector Machine

The support vector machine is a widely used kernel-based supervised learning methodology which constructs an artificial neural network to nonlinearly project its input data onto a high-dimensional space to make an optimal hyperplane as the classification decision. The support vector machine training procedure follows the Vapnik-Chervonenkis dimension theory to optimize the neural network with the minimum structural risk [23]. The most informative data are searched in the mapped space to form the support vectors with the purpose of using several slack variables to make the nonseparable patterns linearly separable, and the decision hyperplane is commonly obtained by maximizing the interclass margin between two classes [24, 25].

In order to compare the classification results, the SVM input features, that is, MDVP:F0, Spread1, MDVP-LDA, Shimmer-LDA, and Nonlinear-LDA, were identical to the inputs of the generalized logistic regression analysis model. In the present work, the input features were mapped by the nonlinear kernels in terms of radial basis function with the spread parameter *σ* = 1 in the high-dimensional space. The support vector machine objective function can be written by combining some equality constraints under the Kuhn-Tucker conditions; then the optimal parameters of the support vector machine model can be derived by solving a nonlinear programming problem [24].

#### 3.2.3. Bagging Classifier Ensemble

The Bagging algorithm is one of the most prevailing ensemble learning paradigms for pattern recognition applications [26]. The Bagging ensemble paradigm commonly contains the procedures of bootstrap sampling and aggregation. Given a set of training data, the Bagging method repeatedly generates a new training set, the size of which is the same as that of the original training data for each based classifier. Some original data instances will appear once again in each generated training set, such that they will replace those absent instances. In the present study, we used 50 decision trees as based classifiers which were trained by the bagged data instances. The outputs of these decision trees were finally aggregated by majority voting for the consensus class labels. The Bagging ensemble generalization error was also estimated with the increase of bagged decision trees.

### 3.3. Classification Evaluation Metrics

We used 5-fold cross-validation technique to test the generalization capability of each classification method. The cross-validation technique first divided the entire set of 195 vocal instances into 5 disjoint subsets (i.e., 39 instances in each subset). In each validation procedure, one subset was selected for testing, and the remaining 4 subsets were used for training the classifiers. Such validation steps were carried out repeatedly until all 5 subsets had been tested for pattern classifications.

The classification results were computed with the confusion matrix metric, in terms of true positive (TP), true negative (TN), false positive (FP), and false negative (FN). Based on the confusion matrix, the parameters of overall accuracy, sensitivity, and specificity were calculated as

(6) Accuracy=TP+TNTP+FP+TN+FN,Sensitivity=TPTP+FN,Specificity=TNFP+TN.

We also computed the Matthews correlation coefficient (MCC) [27] to evaluate the binary classification quality. The merit of the MCC metric is that it incorporates the true and false positives and negatives as a balanced measure between the predicted and actual binary classes. The MCC can be derived from the confusion matrix in the form of correlation coefficient written as

(7) MCC=TP×TN−FP×FNTP+FPTP+FNTN+FPTN+FN.

Similar to other correlation coefficients, the MCC returns a value between −1 and 1. A perfect class agreement outcome produces a value of MCC equal to 1, and MCC > 0 indicates an appropriate binary class prediction. A zero MCC value reveals that the class prediction is no better than random guess. If the MCC value is smaller than 0, it shows that the poor class prediction is even worse than random guess, and a disastrous overall disagreement leads to a MCC value of −1.

In addition, the ROC graphs were generated for visualizing and evaluating binary classification performance. The optimal cutoff point of the ROC curve for the best class prediction of each classification method was chosen in accordance with the maximum Youden's index value (Yindex) [28], that is,

(8) $$\begin{array}{c} \underset{}{\max}\text{Yindex}=Sensitivity+Specificity-1. \end{array}$$

Areas under the ROC curve (AUC) were calculated to measure the effectiveness of the class predictions for three classification methods. As recommended by Demsar [29], the Wilcoxon signed ranks hypothesis test was applied to compare the distinguished vocal patterns of different classification methods in statistical sense (statistically significance: *p* < 0.05).

## 4. Results and Discussion

According to the correlation coefficient values listed in Table 3, the strong linear correlated features (correlation coefficient > 0.8) were associated with fundamental frequency (jitter) perturbations and the noise-to-harmonics ratio.  Table 4 shows the strong correlations among the amplitude perturbation (shimmer) features. Moreover, the nonlinear vocal features of detrended fluctuation scaling index and pitch period entropy are also highly correlated, with the Pearson correlation coefficient of 0.9624. In order to avoid the effects of feature similarity, we used the linear discriminant analysis (LDA) method to project the highly correlated feature dimensions onto the most principal dimension based on linear combination coefficients. The principal dimension of the MDVP:Jitter(%), MDVP:Jitter(Abs), MDVP:RAP, MDVP:PPQ, Jitter:DDP, and NHR features, denoted as MDVP-LDA, was projected by the linear combination coefficients of 0.0062, 4.4 ×10^−5^, 0.0033, 0.0034, 0.0099, and 0.0248, respectively. Then, the principal dimension of the MDVP:Shimmer, MDVP:Shimmer(dB), Shimmer:APQ3, APQ5, APQ11, and Shimmer:DDA features, denoted as Shimmer-LDA, was linearly combined with the coefficients of 0.0297, 0.2823, 0.0157, 0.0179, 0.0241, and 0.047, respectively. The principal dimension of detrended fluctuation scaling index and pitch period entropy features, denoted as Nonlinear-LDA, was computed with the linear combination coefficients of −5.6844 and 0.2066, respectively.

**Figure 1:**
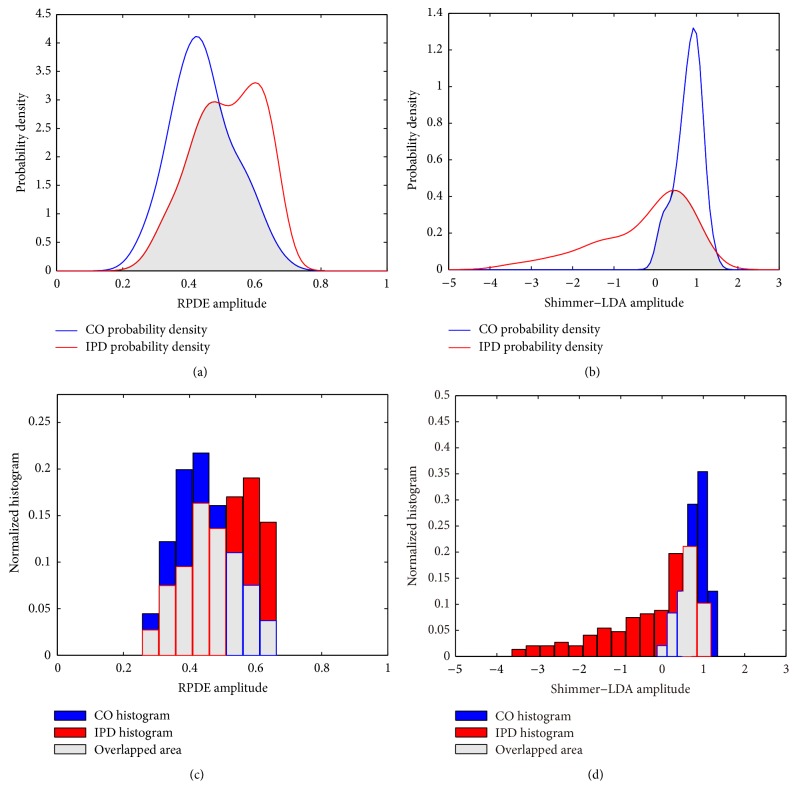
Estimated probability density functions of (a) recurrence period density entropy measure (RPDE) and (b) Shimmer-LDA features, plotted with blue and red curves for healthy control (CO) and idiopathic Parkinson's disease (IPD) subjects, respectively. The overlapping interclass probability risk (ICPR) areas are shown in gray color. Normalized histograms of (c) RPDE and (d) Shimmer-LDA features are also provided with the overlapped areas in gray color, for the purpose of comparison.

Figure 1 illustrates the estimated probability densities and histograms of the Shimmer-LDA and recurrence period density entropy features, respectively. The numbers of histogram bins for the CO and IPD groups were determined according to the Scott's optimal choice rule [30, 31]. The probability density curves are plotted in blue and red colors for healthy control subjects and IPD patients, respectively. It can be observed that the probability density curves are very smooth by using the Parzen-window estimate method, and the RPDE probability distribution curve for IPD patients exhibits multimodality which is quite different from the probability density curve of normal control subjects. According to the probability density curves, it seems that the mean RPDE value of IPD patients is larger than that of normal control subjects, but the Shimmer-LDA variance of normal control subjects is much greater than that of the IPD patients. The ICPR value of each feature is the integration of the overlapped probability density area between the curves of IPD and control subjects, which presents the class-confusion probability with the given feature. The estimated probability density curves and the ICPR areas are similar in all candidate features given in Table 5.

Details of the ICPR and MKLD feature selection results are listed in Table 5. The features with ICPR values < 0.6 imply that the class-confusion probabilities of these features are below 0.6, and the classification error rates are lower than 0.3 with the optimal discrimination of the Bayes decision rule. The features with MKLD values > 1 indicate that the differences of probability density curves are larger than 0.5 between the healthy control and IPD subjects, which would make the classifiers perform better than random guess. Both of the ICPR and MKLD method select five dominant features, with the only difference that the ICPR method selected Spread1 instead of MDVP:Flo, which was chosen by the MKLD method. It is worth noting that the MDVP-LDA, Shimmer-LDA, and Nonlinear-LDA, have manifested distinguishable information, because all these three features are involved in the dominant feature set.

**Figure 2:**
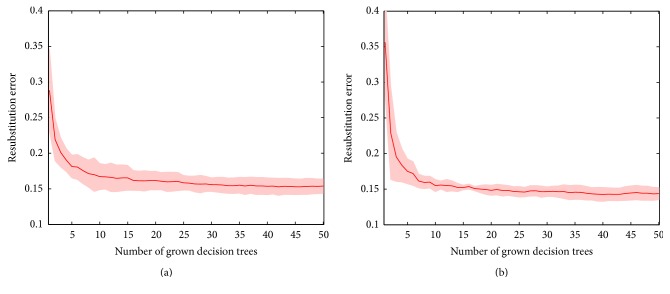
Error curves of the Bagging ensemble input with the (a) modified Kullback-Leibler divergence (MKLD) and (b) interclass probability risk (ICPR) selected features. Boundary of 95% confidence interval is shown in pseudo-red-color.

The resubstitution errors of the Bagging ensemble with the increase of decision trees, based on the MKLD and ICPR input features, are shown in Figure 2, respectively. It can be observed that, with the increasing number of decision trees as base learners, the Bagging ensemble prediction errors are consistently decreasing and finally become convergent.

**Figure 3:**
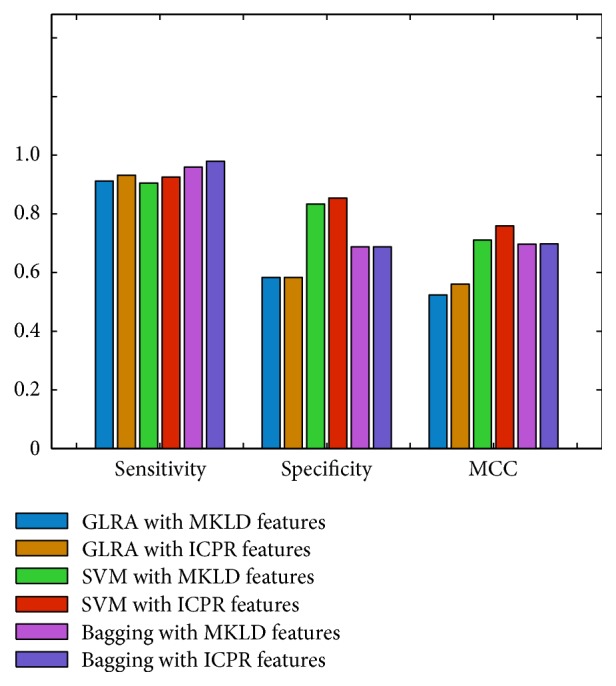
Classification results of the generalized logistic regression analysis (GLRA), support vector machine (SVM), Bagging ensemble, and input with the MKLD and ICPR selected features, respectively. GLRA-MKLD: sensitivity: 0.9116; specificity: 0.5833; MCC: 0.5232; GLRA-ICPR: sensitivity: 0.932; specificity: 0.5833; MCC: 0.5604; SVM-MKLD: sensitivity: 0.9048; specificity: 0.8333; MCC: 0.7105; SVM-ICPR: sensitivity: 0.9252; specificity: 0.8542; MCC: 0.7592; Bagging-MKLD: sensitivity: 0.9592; specificity: 0.6875; MCC: 0.6964; Bagging-ICPR: sensitivity: 0.9796; specificity: 0.6875; MCC: 0.6977.

Figure 3 plots the classification results of the generalized logistic regression analysis, support vector machine, and Bagging ensemble methods with the MKLD and ICPR input features. With the MKLD and ICPR selected features, the generalized logistic regression analysis classifier successfully distinguished 83.08% (sensitivity: 0.9116; specificity: 0.5833) and 84.62% (sensitivity: 0.932 and specificity: 0.5833) vocal patterns, respectively. The generalized logistic regression analysis classifier with ICPR features (Matthews correlation coefficient, MCC: 0.5232) may correctly identify 3 more IPD vocal patterns, which is slightly better than that with MKLD features (MCC: 0.5604).

The support vector machine classification results are much better, with the accurate rates of 88.72% (with MKLD features) and 90.77% (with ICPR features), respectively. The support vector machine has successfully identified 133 (MKLD sensitivity: 0.9048) and 136 (ICPR sensitivity: 0.9252) with the MKLD and ICPR selected features, respectively. It is clear that the ICPR features could help both of generalized logistic regression analysis and support vector machine classifiers better distinguish IPD vocal patterns. In addition, the support vector machine has the major advantage when dealing with control vocal patterns, by providing the high specificity results of 0.8333 and 0.8542 input with the MKLD and ICPR features, respectively.

The Bagging ensemble algorithm provides the accurate classification rates of 89.23% (with MKLD features) and 90.77% (with ICPR features), respectively. The Bagging ensemble algorithm has the superiority with the high IPD vocal pattern identification rates, by providing the sensitivity results of 0.9592 (141 correct IPD cases with MKLD features) and 0.9796 (144 correct IPD cases with ICPR features), respectively. The specificity value of the Bagging ensemble algorithm is 0.6875 (with either MKLD or ICPR features), which indicates that the Bagging ensemble can outperform the generalized logistic regression analysis classifier in detecting healthy control patterns but is still inferior to the support vector machine. The Wilcoxon signed ranks test results show that the support vector machine classifier is significantly superior in classification performance to either the Bagging ensemble algorithm (*p* = 0.037 with the ICPR features and *p* = 0.034 with the MKLD features) or the generalized logistic regression analysis classifier (*p* = 0.017 with the ICPR features and *p* = 0.019 with the MKLD features). However, the classification results of the Bagging ensemble algorithm are slightly better but without a statistical significance (*p* = 0.232 with the ICPR features and *p* = 0.305 with the MKLD features) than those of the generalized logistic regression analysis classifier.

**Figure 4:**
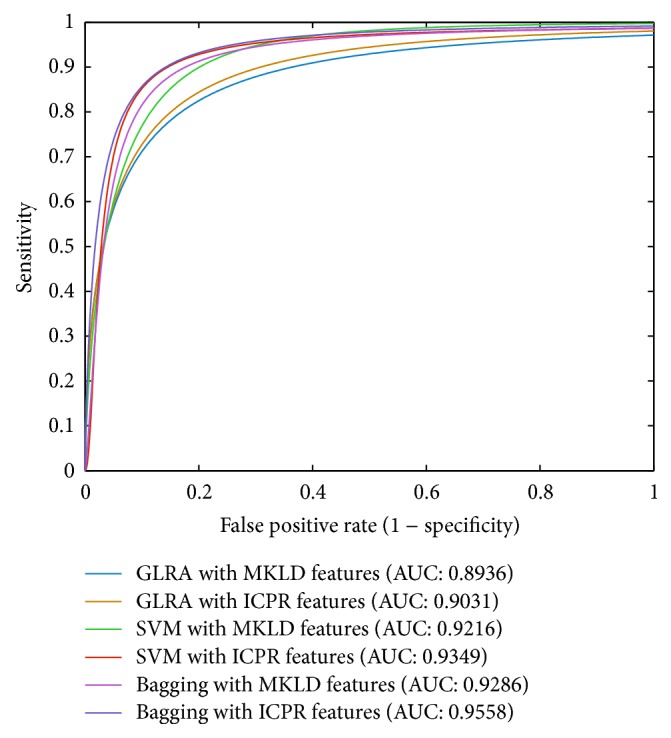
Receiver operating characteristic (ROC) curves and area under the ROC curve (AUC) results of the generalized logistic regression analysis (GLRA), support vector machine (SVM), and Bagging ensemble input with the MKLD and ICPR selected features, respectively. GLRA-MKLD AUC ± standard error (SE): 0.8936 ± 0.024; GLRA-ICPR AUC ± SE: 0.9031 ± 0.0232; SVM-MKLD AUC ± SE: 0.9216  ±  0.023; SVM-ICPR AUC ± SE: 0.9349 ± 0.0219; Bagging-MKLD AUC ± SE: 0.9286 ± 0.0226, Bagging-ICPR AUC ± SE: 0.9558 ± 0.0147.

Concerning the overall classification performance, although the MCC results (MKLD MCC: 0.6964, ICPR MCC: 0.6977) of the Bagging ensemble algorithm are lower than those of the support vector machine (MKLD MCC: 0.7105, ICPR MCC: 0.7592), the Bagging ensemble may output the best ROC curves and the largest area under the ROC curve (AUC) values (MKLD AUC: 0.9286, ICPR AUC: 0.9558) in comparison with the generalized logistic regression analysis (MKLD AUC: 0.8936, ICPR AUC: 0.9031) and support vector machine (MKLD AUC: 0.9216, ICPR AUC: 0.9349), as shown in Figure 4. In general, it is clear from Figure 3 that classification results, in terms of accuracy, sensitivity, specificity, and MCC, of the generalized logistic regression analysis, support vector machine, and Bagging ensemble based on the ICPR selected features are superior to those input with the MKLD selected features. Such results demonstrate the merits of our proposed ICPR feature selection method for IPD vocal pattern analysis. The Bagging ensemble algorithm is very good at identifying IPD vocal patterns with the highest sensitivity results, and the support vector machine is suited for detecting the normal control vocal patterns with the best specificity values. Because the support vector machine is more sensitive to the normal control patterns, the support vector machine is able to provide the highest MCC values among the three classification methods. According to the ROC curves shown in Figure 4, it can be observed that the Bagging ensemble algorithm can provide the best discriminant performance for diagnostic decision making, because the AUC values of the Bagging ensemble algorithm with the MKLD and ICPR features are consistently higher than any other results of the generalized logistic regression analysis or support vector machine.

We analyzed the vocal patterns commonly misidentified by the three classification methods. The misidentified normal control and IPD voices were recorded from six subjects. The healthy subjects are two females both at the age of 66 (subject ID: S42 and S50) and a male aged 69 years (subject ID: S49). Three IPD patients are younger (S02: male, 50 years old, MHAY: 1; S26: male, 53 years old, MHAY: 2; S32: male, 60 years old, MHAY: 2) and with the mild MHAY stage of 1-2, which makes detecting the pathological voice patterns more difficult. It may be interpreted that patients of the mild IPD disease stage are more likely to produce normal voice sounds, in comparison with those with severe stages.

Random forest is an extension of Bagging ensemble by randomly selecting feature subspace to train base learners with split feature subsets and combine their outputs for ensemble predictions [32]. The reason why we chose the Bagging ensemble instead of random forest is that we would like to evaluate the effectiveness of our feature selection and analysis method. The same feature combination inputs for the three machine learning methods also make the classification results comparable. In the present work, the Bagging ensemble along with the ICPR selected features provided a higher AUC value than that of the maximum* a posteriori* (MAP) decision rule (AUC: 0.94) in our previous work [5]. The support vector machine input with the ICPR features also produced better ROC curve results than the support vector machine input with the kernel principal components analysis features (AUC: 0.85) reported in our previous work [5]. The classification results of the Bagging ensemble with ICPR features only trained by a relative small data set of 195 voice records are also comparable to the results (accuracy: 91.8%, sensitivity: 0.954) with a much larger data set of 707 voice records reported by Little et al. [4].

The present work also has some limitations. The correlations between a number of vocal parameters limit the performance improvement of classifiers. We only studied the linear correlations in the present work. But it is believed that some nonlinear correlation analysis methods could be considered in the next step of related works. It is noted that the average age of the CO group is 7.6 years younger that of the IPD group and the aging factor could more or less affect the voice quality. However, the current voice data set with a relative small size (195 records) limits the further study of aging effects in analysis of vocal parameters and patterns, as well as the effectiveness of three classifiers. Classification performance of the Bagging ensemble still needs to be evaluated with a much larger data in the future work.

## 5. Conclusions

Quantitative analysis of pathological voice is very useful in the clinical applications of dysphonia detection and therapy assessment of phonation system. In the present work, we proposed the ICPR feature selection method by selecting the features of lower interclass overlapping feature probability risks. The features selected by the ICPR criterion are MDVP:F0, Spread1, MDVP-LDA, Shimmer-LDA, and Nonlinear-LDA, which involve the major fundamental frequency measures, amplitude variations, and nonlinear parameters of the vowel sounds. The experimental results showed that the generalized logistic regression analysis, support vector machine, and Bagging ensemble methods with the ICPR features can perform better than with the MKLD features. The classification results suggest that the support vector machine and Bagging ensemble methods can effectively identify healthy control and IPD vocal patterns with high overall accurate rates and MCC values and excellent ROC curves for diagnostic decision making. For the future related works, new acoustic signal dynamic parameters and some state-of-the-art machine learning methods [33], such as convolutional neural networks and recurrent neural networks, could also be positively considered to improve the classification performance.

## Figures and Tables

**Table 1:** Pathological stages of 23 patients with Parkinson's disease rated by the modified Hoehn and Yahr (MHAY) scale.

MHAY stage	Disease description	Number of patients
1	Minimal functional involvement	3
1.5	Unilateral involvement	3
2	Bilateral involvement	5
2.5	Mild bilateral disease	7
3	Mild to moderate bilateral disease	4
4	Severe functional disability	1

**Table 2:** Voice perturbation and nonlinear dynamic parameters measured from the acoustic signals of 31 subjects [5, 7].

Abbreviations	Feature description
MDVP:F0 (Hz)	Average vocal fundamental frequency
MDVP:Fhi (Hz)	Maximum vocal fundamental frequency
MDVP:Flo (Hz)	Minimum vocal fundamental frequency
MDVP:Jitter(%)	MDVP jitter in percentage
MDVP:Jitter(Abs)	MDVP absolute jitter in ms
MDVP:RAP	MDVP relative amplitude perturbation
MDVP:PPQ	MDVP five-point period perturbation quotient
Jitter:DDP	Average absolute difference of differences between jitter cycles
MDVP:Shimmer	MDVP local shimmer
MDVP:Shimmer(dB)	MDVP local shimmer in dB
Shimmer:APQ3	Three-point amplitude perturbation quotient
Shimmer:APQ5	Five-point amplitude perturbation quotient
MDVP:APQ11	MDVP 11-point amplitude perturbation quotient
Shimmer:DDA	Average absolute differences between the amplitudes of consecutive periods
NHR	Noise-to-harmonics ratio
HNR	Harmonics-to-noise ratio
RPDE	Recurrence period density entropy measure
D2	Correlation dimension
DFA	Signal fractal scaling exponent of detrended fluctuation analysis
Spread1	Two nonlinear measures of fundamental
Spread2	Frequency variation
PPE	Pitch period entropy

**Table 3:** Pearson correlation coefficients between the vocal features of MDVP:Jitter(%), MDVP:Jitter(Abs), MDVP:RAP, MDVP:PPQ, Jitter:DDP, and NHR.

Features	MDVP:Jitter(%)	MDVP:Jitter(Abs)	MDVP:RAP	MDVP:PPQ	Jitter:DDP	NHR
MDVP:Jitter(%)	1	0.9357	0.9903	0.9743	0.9903	0.907
MDVP:Jitter(Abs)	0.9357	1	0.9229	0.8978	0.9229	0.835
MDVP:RAP	0.9903	0.9229	1	0.9573	1	0.9195
MDVP:PPQ	0.9743	0.8978	0.9573	1	0.9573	0.8446
Jitter:DDP	0.9903	0.9229	1	0.9573	1	0.9195
NHR	0.907	0.835	0.9195	0.8446	0.9195	1

**Table 4:** Pearson correlation coefficients between the vocal features of MDVP:Shimmer, MDVP:Shimmer(dB), Shimmer:APQ3, Shimmer:APQ5, MDVP:APQ11, and Shimmer:DDA.

Features	MDVP:Shimmer	MDVP:Shimmer(dB)	Shimmer:APQ3	Shimmer:APQ5	MDVP:APQ11	Shimmer:DDA
MDVP:Shimmer	1	0.9873	0.9876	0.9828	0.9501	0.9876
MDVP:Shimmer(dB)	0.9873	1	0.9632	0.9738	0.961	0.9632
Shimmer:APQ3	0.9876	0.9632	1	0.9601	0.8966	1
Shimmer:APQ5	0.9828	0.9738	0.9601	1	0.9491	0.9601
MDVP:APQ11	0.9501	0.961	0.8966	0.9491	1	0.8966
Shimmer:DDA	0.9876	0.9632	1	0.9601	0.8966	1

**Table 5:** Feature selection by means of the interclass probability risk (ICPR) and modified Kullback-Leibler divergence (MKLD) methods. Bold values are selected features for pattern classifications (ICPR < 0.6; MKLD > 1).

Features	ICPR	MKLD
MDVP:F0	**0.59**	**1.02**
MDVP:Fhi	0.71	0.26
MDVP:Flo	0.67	**1.13**
HNR	0.66	0.73
RPDE	0.71	0.25
D2	0.76	0.22
Spread1	**0.56**	0.89
Spread2	0.69	0.46
MDVP-LDA	**0.59**	**1.96**
Shimmer-LDA	**0.46**	**5.72**
Nonlinear-LDA	**0.43**	**2.17**
